# Connectivity patterns in the DMN that are impacted by traumatic stress

**DOI:** 10.21203/rs.3.rs-9544648/v1

**Published:** 2026-07-03

**Authors:** Dayan Knox, Negin Mohammadmirzaei, Simone Lunn, Praveen Kulkarni, Matthew Biddle, Alyssa Burlack, Aryan Mehta, Emma Lopes, Alyssa Gunning, Khan Hekmatayar

**Affiliations:** University of Delaware; University of Delaware; University of Delaware; Dana-Farber Cancer Institute; University of Delaware; University of Delaware; University of Delaware; University of Delaware; Georgia State University

## Abstract

Decreased connectivity within the default mode network (DMN) has been consistently implicated in post traumatic stress disorder (PTSD), but critical nodes through which traumatic stress changes DMN connectivity and DMN connectivity patterns that are linked to specific traumatic stress effects are not well understood. To address this, resting-state functional connectivity (rs-FConn) within the DMN was analyzed using modular and graph theory tools to characterize DMN connectivity changes brought on by single prolonged stress (SPS); a rat traumatic stress model. Results identified a set of negative edges that connect anterior and posterior DMN nodes. We refer to these as A-P edges and the anterior cingulate cortex (ACC) and rostral retrosplenial cortex (rRSC) were nodes that had the largest number of these edges. A-P edge frequency and graph connectivity measures decreased with a second fMRI scan in control rats and these decreases, and rRSC A-P edge frequency, were disrupted by SPS. Traumatic stress leads to deficits in extinction retention and to examine how DMN connectivity changed during extinction, we used correlated c-Fos levels among select DMN nodes to approximate DMN connectivity during fear/threat conditioning, and extinction learning and memory. Results suggest that SPS decreased DMN connectivity under most conditions, but enhanced DMN connectivity during extinction testing. Overall, the results of this study raise the possibility that while SPS does decrease DMN connectivity and disrupts changes in DMN connectivity brought on by a second fMRI scan, certain aspects of DMN connectivity are enhanced with SPS.

## 1. Introduction

Post traumatic stress disorder (PTSD) is a debilitating psychiatric disorder characterized by clusters of symptoms (1, 2). Given the economic burden of PTSD (3), examining neurobiological processes through which traumatic stress leads to specific PTSD symptoms is significant. To this end, animal models can be useful for establishing relationships among stress effects and neural activity.

In vivo MRI technology has been used in clinical studies to identify functional circuits that are impacted by traumatic stress (4–15) and have revealed that PTSD is associated with decreased resting state functional connectivity (rs-FConn) within the default mode network (DMN) (4, 14, 16, 17). However, DMN changes that occur as a result of traumatic stress is difficult to determine, because very few studies include pre-trauma data. Yet, having this data can allow for characterization of the impact of traumatic stress. For example, clinical studies that included pre-trauma fMRI data has shown that changes in coupling between the salience network and DMN can predict perceived stress that resulted from repeated emergency responder events (18). Using the single prolonged stress (SPS) model of traumatic stress (a validated rodent model (19–23)) we have previously shown that SPS disrupts DMN connectivity, and may do so by decreasing how the retrosplenial cortex (RSC) and anterior cingulate cortex (ACC) are integrated with other DMN nodes (24). However, RSC and ACC connectivity patterns that are impacted by SPS remain unknown. Also unknown is DMN connectivity patterns that are affected by SPS and associated with specific SPS effects.

**Figure 1:**
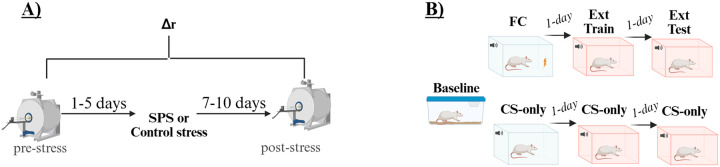
Experimental Design. A) Rs-FConn changes from pre-stress to post-stress scans (i.e. Δr) were compared for DMN edges. B) We also correlated c-Fos levels between select DMN nodes at baseline, fear/threat conditioning, extinction training, extinction testing; or during novel and repeated tone-CS presentation (CS-only condition). This was done to approximate how DMN connectivity changed during these behavioral sessions.

To address these issues we performed rs-FConn in animals prior to and after the application of SPS. We then used modularity and graph analytical tools to characterize how SPS changed DMN connectivity. The DMN is characterized by correlated BOLD activity when an organism is not engaging external stimuli (25–29). DMN connectivity is often measured using correlated BOLD signals, but other techniques have been used to approximate DMN connectivity (e.g. EEG) (30–34). Because using fMRI to measure DMN connectivity in rats is typically accomplished under light anesthesia or in head-fixed animals, using different modalities to approximate DMN connectivity would allow for monitoring of approximate DMN connectivity across a variety of behavioral conditions. Here we used a database of c-Fos levels under baseline, threat/fear conditioning, and extinction training and testing. We used correlations between critical DMN nodes as an approximate measure of DMN connectivity during the behavioral sessions. The experimental design is illustrated in Fig. 1.

## 2. Material and Methods

### 2.1 Animals

Thirty-one (n = 15 male and n = 16 female) Sprague-Dawley rats were used for the rs-FConn analyses and a database that comprised 134 male Sprague-Dawley rats were used for correlational analyses involving c-Fos. Rats weighted approximately 150–250 g upon arrival and were obtained from Charles River Inc. The male and female rats were housed separately in dedicated rooms. Upon arrival, the rats were housed in same sex pairs in a temperature and humidity controlled room with a 12-hour light/dark cycle and ad libitum access to food and water. A minimum acclimatization period of three days was allowed before initiating any testing. All experimental procedures were reviewed and approved by the University of Delaware Institutional Animal Care and Use Committee in compliance with NIH guidelines.

### 2.2 SPS

SPS or control stress was conducted as previously described (19, 35–37). SPS consisted of 120 minutes of restraint, 20 minutes of forced swim, and ether exposure until general anesthesia was induced. Control rats were relocated to a new room in their home cages for the equivalent duration of SPS. After undergoing stress protocols, rats were returned to their respective rooms and individually housed with free access to food and water. Prior to experimental testing, a 7–10 day post-stress incubation period was allowed to elapse, as this duration is necessary to observe the effects of SPS (19, 36–41).

### 2.3 Experimental design and fMRI data acquisition

The experimental design is illustrated in Fig. 1A. Three to five days after acclimation (and prior to any stress protocols), rats were brought to a 9.4T Biospec Brucker (Billerica MA) scanner in order to perform rs-fMRI scans. Each animal was put under general anesthesia using isoflurane (5% in oxygen), given a subcutaneous injection of .1 ml of a 2% lidocaine solution on the scalp to alleviate any discomfort, and placed within the MRI cradle in the head prone position. Animals were then maintained under general anesthesia using 1–1.5% isoflurane in oxygen through a nose cone. This level of anesthesia coupled with respiration aid is associated with comparable BOLD signals to awake animals that undergo rs-fMRI (26, 42–47). Throughout the procedure, respiration and body temperature were continually monitored and sustained at 60–80 breaths per minute and 34–36°C, respectively.

Rs-fMRI scans were acquired over a period of 12 min and 34 sec using a multi-slice Half Fourier Acquisition Single Shot Turbo Spin Echo (HASTE) pulse sequence with the following parameters: repetition time (TR)/echo time (TE) = 1256 ms/18 ms, field of view (FOV) = 2.88 × 2.88 cm^2^, matrix size = 96×96 [0.3-mm in-plane resolution], slice thickness = 1.0 mm, 25 slices, and 200 repetitions. One to five days after the pre-stress scan, rats were subjected to SPS or control stress. 1–3 days after the post-stress incubation period of SPS, a rs-fMRI scan was performed in all animals for a second time using the protocols described above.

### 2.4 Experimental design and c-Fos data acquisition

We have generated a database of c-Fos expression across emotional and sensory brain regions at baseline, fear/threat conditioning, extinction training, and extinction testing (Fig. 1B). As a control we performed the same protocol in a group of rats that were presented with tone-CS only presentations only during fear/threat conditioning (i.e. no footshock or unconditioned stimulus presentation). This served as a control for repeated tone-CS presentation. We have previously published findings from this dataset (48–50). In this study, we correlated c-Fos levels between a subset of brain regions from the DMN to approximate how DMN connectivity changed at baseline, fear/threat conditioning, extinction training, and extinction testing in SPS and control male rats.

Details of the protocols used to generate this database can be found elsewhere (48–50) and we only briefly describe them here. Animal were either fear conditioned (co-terminating five auditory CSs (10s, 2kHz) and footshocks (1s, 1mA) with 60s ISIs) or presented with just auditory CSs only. Extinction training was 30 CS presentations and extinction testing was 10 CS presentations, both in the absence of footshocks. We then measured c-Fos levels in subsets of rats that were euthanized after fear/threat conditioning, extinction training, extinction testing, or immediate removal from the housing colony to establish baseline levels of c-Fos. To measure c-Fos levels rats were rapidly euthanized, brains extracted, and stored in a −80 C freezer. Thirty micron brain sections were sliced using a cryostat (CM1350 Leica Inc), mounted to superfrost slides then subjected to immunohistochemical protocols to label c-Fos in multiple brain regions using methods previously described by us (48, 50, 51). We have previously demonstrated the specificity of this protocol to measure protein expression of interest (48–50, 52).

### 2.5 MRI processing

ImageJ software was used to perform skull stripping, then a bash shell script integrating essential tools such as FSL, AFNI, and ANTs, facilitated the preprocessing and analysis of our fMRI data. Image preprocessing encompassed motion correction, despiking, slice-timing correction, low BOLD frequency filtering, and volume registration. Mean time series were computed for regions of interest (ROIs encompassing 174 nodes), and Pearson correlation (rs) were calculated between the time series data from different ROIs. The r-values were then used within MATLAB to generate a 174×174 matrix, visually illustrating the connectivity patterns among the 174 brain nodes in each rat.

### 2.6 Modular analysis of DMN

The rat DMN was subjected to modular analysis as previously described (25) for the prestress, post-control, and post-SPS scans. All edges (i.e. r-values) that comprise the DMN from pre-stress (n = 31), post-SPS (n = 16), and post-control (n = 15) scans were subjected to a one-sample t-test (test value = 0) with Bonferroni corrections applied (p < .0005). We did not separate male and female rats for this analysis, because we previously observed that SPS changes in DMN connectivity are equivalent in male and female rats (53). Edges that survived statistical significance were used to define the modular-DMN (mod-DMN) for the prestress, post-control, and post-SPS scans. The resulting matrices were then analyzed in Cytoscape to determine graph characteristics (e.g. diameter, path length) and modularity using the Cytocluster app and the ClusterOne algorithm (54). For modular analyses, a minimum of three nodes per module was required with r-values between nodes serving as weights for the edges. For these analyses a p < .05 was used as a criterion for statistical significance.

To determine if the scalar property of r-values (range 0–1) in mod-DMNs changed with SPS, we subjected r-values among select nodes (determined by the results of the modular analysis) to factor designs that involved stress (SPS vs. control) and time (e.g. pre-stress vs. post-stress). Main and simple effects were analyzed using analysis of variance (ANOVA), while main and simple comparison were analyzed using t-test with Bonferroni corrections applied where appropriate. For these analyses a p < .05 was used as a criterion for statistical significance.

### 2.7 C-Fos approximation of DMN connectivity

We used a subset of nodes from the mod-DMNs to generate approximate DMN connectivity from correlated c-Fos levels among brain regions. The exact nodes used for this analysis depended on critical nodes identified from the modular analyses. C-Fos levels during behavioral sessions (e.g. baseline, fear/threat conditioning, extinction training, extinction testing) within nodes were subjected to correlations that were corrected using B-H corrections (55) (p < .05, FDR < .05). It is important to note that this was a correlation between an end measure of neural activation (i.e. c-Fos), and not moment to moment changes in a measure of neural activity (e.g. BOLD). We used correlated c-Fos levels to approximate DMN connectivity across multiple behavioral conditions. Baseline levels of c-Fos within a node was analyzed using t-test (SPS vs. control). C-Fos levels in nodes of the DMN were normalized relative to baseline levels and subjected to a stress (SPS vs. control) × condition (CS-fear vs. CS-only) × trial type (Fear/threat conditioning, Extinction training, Extinction testing) factor design. Main and simple effects were analyzed using ANOVA, while main and simple effects were analyzed using t-test with Bonferroni corrections applied where necessary.

## 3. Results

### 3.1 Impact of SPS on DMN connectivity

**Figure 2:**
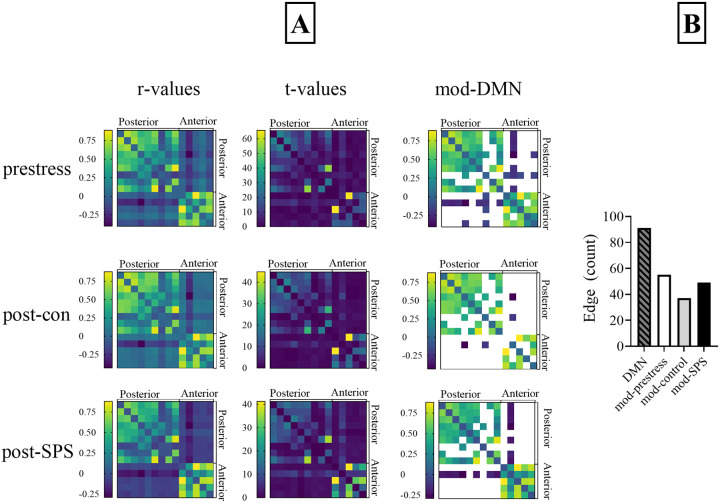
A) Heatmaps showing r-values, t-stats, and surviving edges of the prestress, post-Control, and post-SPS DMNs. B) After applying statistical test, the number of possible edges in the DMN decreased in all modular DMNs.

Verification of correct brain registration and imaging can be found in a previous publication of a subset of this fMRI dataset (53). Figure 2A shows averaged r-values for all possible edges within the DMN, t-values for one-sample t-tests, and edges that survived statistical analysis. As expected, there was a decrease in edge frequency (with respect to all possible edges within the DMN) in all mod-DMNs (i.e. prestress, post-control, post-SPS) (Fig. 2B).

**Figure 3:**
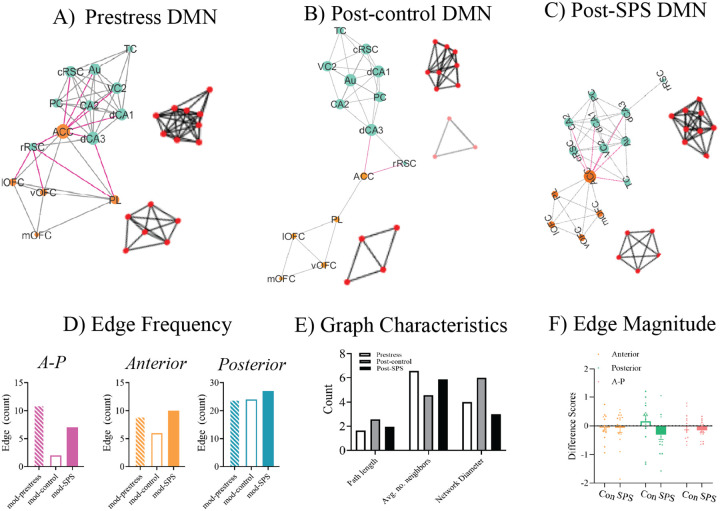
Modular and graph analysis of DMN across imaging conditions. A) The prestress DMN had an anterior and posterior pole as identified by modular analysis (modules shown next to graph in red nodes). The ACC was part of the anterior and posterior pole and even though the rRSC is within the posterior DMN, it was grouped with the anterior module because of its extensive edges with anterior modules. All anterior-posterior modules were connected with negative edges (i.e. inverse correlations, shown in pink) and the ACC, rRSC, and dCA3 had these edges (i.e. A-P edges). B) The post-Control DMN had anterior and posterior modules, but none of these modules involved the ACC, rRSC, or dCA3. Also the number of A-P edges decreased with the second fMRI. C) The post-SPS DMN had anterior and posterior modules and A-P edges involving the ACC. The ACC was also grouped in the anterior and posterior module. However, the rRSC was only connected to dCA3 (identified as an outlier) and had no A-P edges. D) Frequency of edge occurrence in the modular DMNs. The frequency of A-P edges decreased in control and SPS animals with a second fMRI. However, anterior edge frequency only decreased in control animals. In SPS animals anterior edge frequency increased. Posterior edge frequency was unchanged in control animals and increased in SPS animals with a second fMRI. E) Measure of graph connectivity in modular DMNs. All measures of DMN connectivity decreased in the post-Control DMN (i.e. path length, average node neighbor, graph diameter). Some measure of DMN connectivity decreased in the post-SPS DMN (e.g. path length, average node neighbor), but graph diameter decreased, which suggest an increase in connectivity among nodes. F) The impact of SPS on the magnitude of connectivity within anterior, posterior, and A-P edges in the DMN. SPS may have decreased the magnitude of posterior and A-P DMN connectivity.

Next we imported the modular prestress, post-Control, and post-SPS DMNs into Cytoscape. Nodes in the anterior DMN are shown in orange and in the posterior DMN, teal. Node size is mapped to the edge frequency of a node. The prestress mod-DMN had an anterior and posterior pole (Fig. 3A). This was revealed by modular analysis that identified a module [density = .861, quality = .838, p < .001] that consisted of nine nodes that are within the posterior pole of the rat DMN, with the exception of the anterior cingulate cortex (ACC). The ACC is within the anterior DMN, but was grouped into the posterior DMN, because of its negative edges (inverse correlations that are shown in pink) with posterior DMN nodes. A second module [density = .867, quality = .619, p = .03] consisted of nodes that are considered within the anterior DMN (including ACC) with the exception of the rostral retrosplenial cortex (rRSC) which is anatomically in the posterior DMN, but was grouped into the anterior DMN, because of its negative edges with anterior nodes (Fig. 3A). Dorsal CA3 (dCA3, posterior node) also had a unique negative edge with an anterior node (prelimbic cortex (PL)). We refer to edges that are negative and connect nodes between the anterior and posterior DMN as A-P edges. The ACC was clustered into the anterior and posterior DMN modules, and was considered an A-P node.

The post-Control mod-DMN is illustrated in Fig. 3B. Modular analyses identified three modules, but only two attained statistical significance. The first module [density = .821, quality = .885 p < .001] consisted of eight nodes within the posterior DMN. The second module [density = .833, quality = .833 p = .012] consisted of four nodes within the anterior DMN. However, the ACC, rRSC, and dCA3 were clustered together, but analysis of this module did not attain statistical significance [density = 1.33, quality = .444 p = .329].

The post-SPS mod-DMN is illustrated in Fig. 3C. Modular analyses identified two modules. The first module [density = .833, quality = .857, p < .001] consisted of nine nodes that are considered part of the posterior DMN, except for the ACC. Analysis of the second module approached significance [density = 1, quality = .588 p = .053] and consisted of five nodes that are considered part of the anterior DMN (including the ACC). The rRSC was only connected to dCA3, this edge was positive, and the rRSC was not considered as part of a module (identified as an outlier). The ACC still had multiple A-P edges and was an A-P node.

We then characterized changes in edge frequency and graph connectivity from prestress to the post-stress mod-DMNs. Results suggest that, in comparison to the prestress mod-DMN, anterior and A-P edges were less frequent in the post-Control DMN, but the occurrence of posterior edges were similar.

For the post-SPS DMN, the occurrence of posterior and anterior edges increased, but the number of A-P edges decreased (Fig. 3D). Graph connectivity measures decreased in the post-Control DMN relative to the prestress DMN. These included an increase in path length (i.e. larger values mean less connected), decrease in average node neighbor, and an increase in graph diameter (larger means less connected). For the post-SPS mod-DMN, path length increased and average node neighbor decreased. However, the graph diameter decreased (i.e. increased connectivity) relative to the prestress DMN. Thus, while there was an overall decrease in connectivity in the post-control DMN, for the post-SPS DMN, some connectivity measures increased, while other measures decreased (Fig. 3E).

To further characterize how connectivity within anterior, posterior, and A-P edges changed with repeated fMRI and SPS, we used nodes and edges from the prestress-DMN to generate mod-DMNs in individual animals prior to and after SPS. We then arranged anterior, posterior, and A-P edges from these graphs into respective vectors, and calculated the length of these vectors, as this can be used to characterize connectivity within the DMN (24). Anterior, posterior, and A-P vector lengths in the post modular DMNs were subtracted from the prestress values to yield difference scores. These difference scores were analyzed using a stress (SPS vs. control) × edge type (A-P, anterior, posterior) factor design. There was a stress × edge type interaction that approached significance [quadratic component: F_(1,28)_ = 3.67, p = .066]. Descriptive analysis raised the possibility that the magnitude of DMN connectivity did not change in control rats, but that posterior and A-P edge magnitude decreased in SPS rats. These results are illustrated in Fig. 3F.

### 3.2 Impact of SPS on approximated DMN connectivity during fear/threat and extinction, learning and memory.

To construct an approximated DMN based on correlated c-Fos levels, we used c-Fos expression in the ACC and rRSC, because these are nodes in different DMN poles (anterior and posterior respectively) that have multiple unique A-P edges. We also included the OFC (averaged across lateral and ventral divisions) and the dCA1, because these nodes are in different DMN poles and have no unique A-P edges. The number of nodes (four in total) also allowed for multiple correlations that could survive FDR corrections given the n/independent group in the database we were probing (see supplemental information).

**Figure 4:**
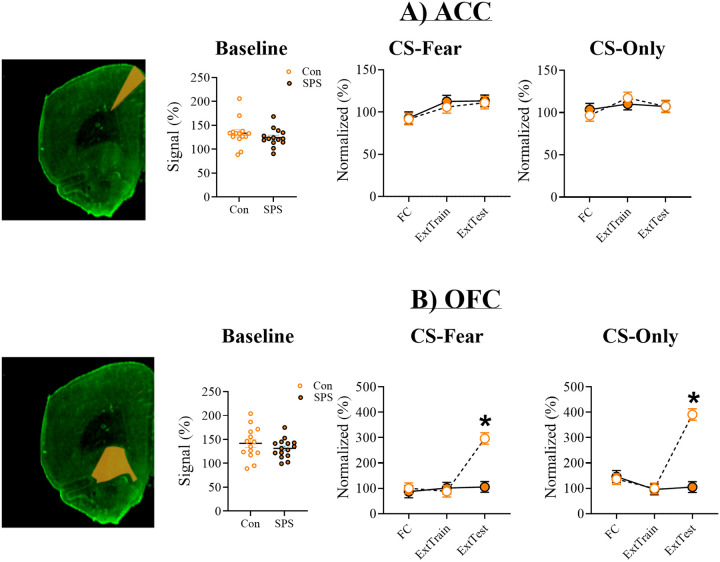
Impact of SPS on c-Fos levels in the ACC and OFC across different behavioral sessions. A) SPS had no impact on c-Fos expression within the ACC across any condition. B) OFC c-Fos expression was enhanced during extinction testing in control rats, but this was attenuated in SPS rats. These effects occurred irrespective of whether animals were initially fear/threat conditioned or presented with CSs only. * - p < .05.

We have previously published the impact of SPS on c-Fos expression in the rRSC and dCA1 (50, 51). As such we only report the impact of SPS on c-Fos levels in the ACC and OFC. SPS had no impact on baseline c-Fos levels in the ACC or OFC and no impact on c-Fos levels in the ACC. OFC c-Fos levels were enhanced in control rats during extinction testing and this enhancement was decreased in SPS animals. This was revealed by a stress × condition interaction [F_(2,88)_ = 39.693, p < .001]. However, this was observed in the CS-fear (fear and extinction memory) and CS-only (repeated CS presentation) groups, as there were no main or interaction effects involving condition (ps > .05) (Fig. 4).

**Figure 5:**
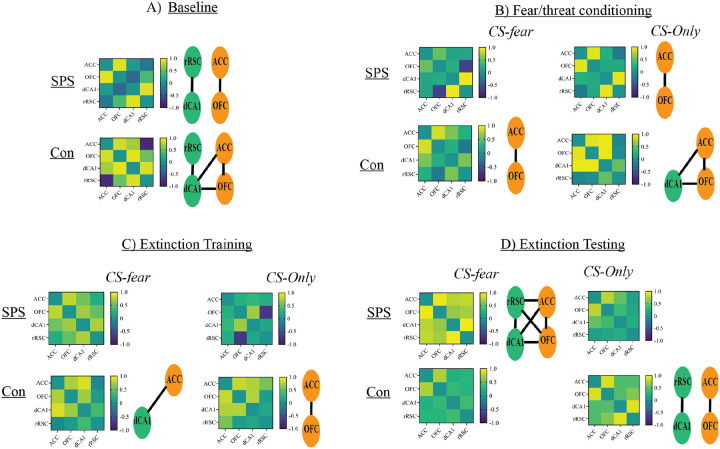
Impact of SPS on connectivity in the approximate-DMN. Heat maps show the connectivity among all nodes within the approximate-DMN. Next to these heat maps are ball and stick figures that show connections between nodes that were significant. Approximate-DMN connectivity at baseline was highest relative to all other behavioral conditions in control rats (A-D). Approximate-DMN connectivity in control rats was higher than connectivity in SPS rats in all conditions (A-C), except extinction testing for animals that were initially fear/threat conditioned (CS-Fear) (D). In this condition, connectivity in the approximate-DMN was maximal within the SPS treatment and higher than control rats.

Figure 5 shows connectivity within approximate-DMNs across the different behavioral sessions. For control rats, connectivity within the approximate-DMN was highest as baseline relative to all other sessions. For SPS rats, connectivity within the approximate-DMN was highest during the extinction testing/CS-Fear session. When comparing SPS and control rats, connectivity within the approximate-DMN of control rats was higher in 11/12 sessions and lower in only the extinction testing/CS-Fear.

## 4. Discussion

The results of the study replicate findings that have examined the rat DMN, but also identifies novel aspects. We observed two modules/poles which is consistent with previous studies (25–27), but also observed inverse correlations that consistently involved the ACC and rRSC. While inverse correlations could result from preprocessing protocols (56) it is unlikely this was the case in this study, because we used standard preprocessing protocols (57) that did not utilize global signal regression, inverse correlations were systematic (involved primarily the ACC and rRSC), and were not invariant (i.e. changed with second fMRI). Also, inverse correlations, while not surviving FDR for c-Fos correlations, were observed at baseline in control rats further supporting the assertion that inverse correlations within the DMN do not necessarily represent artifact.

A previous study has identified the ACC and RSC as central anterior and posterior nodes within anterior and posterior poles, respectively, of the rat DMN (25). In this study the ACC had the most edges with anterior nodes, but also had extensive A-P edges with posterior nodes. The rRSC had mostly A-P edges, but the caudal RSC (cRSC) had edges with mostly posterior nodes. These findings suggest that the ACC and rRSC represent unique nodes within the DMN that could bridge connectivity between the anterior and posterior poles of the DMN, and that the rRSC and cRSC may serve different functions within the DMN.

Connectivity within the DMN is largest when an organism is at rest and/or not engaging external stimuli (28, 29, 34, 58, 59). To the best of our knowledge, this is the first time a decrease in DMN connectivity has been observed when fMRI is performed in an organism for a second time (i.e. repeated fMRI). Interestingly, it is the frequency of edges (mostly A-P edges) that decrease with repeated fMRI and not the magnitude of r-values (i.e. scalar value of edges). A change in the frequency of DMN edges without changes in the magnitude of r-values could mean that there is increased variability within the DMN with repeated fMRI, but further research is needed to examine the cause (or source) of this variability.

In SPS animals, decreases in DMN edge frequency brought on by repeated fMRI was reduced. Furthermore, while measures of graph connectivity did decrease (increased path length, average node neighbor) with repeated fMRI in SPS rats, graph diameter decreased, which suggests some DMN connectivity increased with repeated fMRI in SPS rats. Of interest is the loss of connectivity of the rRSC in SPS animals. Modular analysis identified this node as an outlier, as the rRSC had only a single connection with the dCA3 in the post-SPS DMN. Previous research has shown that SPS decreases baseline c-Fos levels in the rRSC (51) and the rRSC does not cluster into components that contain DMN nodes in SPS rats (24). Unlike control animals, the magnitude of r-values among A-P and posterior edges may have decreased with repeated fMRI in SPS rats. The overall results suggest that repeated fMRI decreases the frequency of anterior and A-P edges in the DMN without changing the magnitude of these edges. SPS disrupts both effects with smaller changes in the anterior and A-P edge frequency and decreased magnitude of posterior and A-P edge magnitude. In this way, and with respect to repeated fMRI, SPS may increase the rigidity of the DMN, while decreasing the magnitude of connectivity.

SPS disrupted approximate DMN connectivity in almost all behavioral sessions. This finding is consistent with the assertion that traumatic stress disrupts DMN connectivity (4, 14, 24). However, SPS enhanced approximate DMN connectivity when presented with an extinguished CS. Enhanced fear/threat conditioned responding during an extinction test is consistently observed with SPS exposure (19, 60–62). The finding that enhanced approximate-DMN connectivity occurred during extinction testing in SPS rats that were initially fear/threat conditioned raises the possibility that this effect could be linked to enhanced conditioned responding during an extinction test. What might enhanced DMN connectivity in SPS rats be indicative of? Episodic memory recall is associated with enhanced DMN connectivity (28, 63–65). Presentation of an extinguished CS has attributes of episodic memory as it involves what (CS that could signal danger or safety), when (either one or two days prior), and where (threat or safety context) of an experience (66). Presentation of the extinguished CS to SPS animals may have resulted in episodic memory recall of the CS when it signaled footshock (e.g. fear/threat conditioning), which was reflected by enhanced DMN connectivity. It is important to note that in control rats that were initially subjected to fear/threat conditioning, enhanced DMN connectivity was not observed during extinction testing. This discrepancy could mean that different processes drive freezing in SPS vs. control rats during an extinction test. A possibility could be that episodic memory recall is invoked with presentation of an extinguished CS in SPS rats, but in control rats threat processing may predominate. These possibilities are speculative, but consistent with the results of this study and what is known about DMN activation. Future research would be needed to examine these possibilities.

During extinction testing c-Fos levels in the OFC were enhanced in control animals and this effect was disrupted in SPS animals. OFC divisions are part of the anterior DMN (25) and may represent nodes through which SPS enhances DMN connectivity (and also freezing) during extinction testing. Activation of the OFC with NMDA can inhibit fear expression (67, 68), which raises the possibility that enhanced OFC activation during an extinction test could contribute to inhibition of conditioned freezing and possibly diminished DMN activation in control rats. However, it should be noted that OFC activation (and the blunting of this effect with SPS) was observed during the extinction test irrespective of whether animals were initially fear/threat conditioned or not. Thus, it is not clear why there was an enhancement of c-Fos expression in the OFC in control rats or why this effect was disrupted with SPS. It is not well understood how changes in neural activity leads to changes in connectivity within the DMN and more research is needed to address this gap.

The results of the study show that the ACC and rRSC represent unique nodes in the DMN where they have A-P edges. The results also show that the frequency of A-P and anterior edges in the DMN decrease with repeated fMRI and SPS disrupts this effect. SPS also decreases the magnitude of connectivity within the DMN (i.e. increased rigidity and decreased magnitude of connectivity). Lastly, while approximated DMN connectivity decreases in SPS animals during multiple conditions, enhanced approximate DMN connectivity is observed if SPS animals are presented with an extinguished CS.

## Supplementary Material

Table 1 is available in the Supplementary Files section.

This is a list of supplementary files associated with this preprint. Click to download.

- supplementarydata.docx
- Table1.docx

## Data Availability

Data from this manuscript will be made freely available upon request
